# CD4-mimetic sulfopeptide conjugates display sub-nanomolar anti-HIV-1 activity and protect macaques against a SHIV162P3 vaginal challenge

**DOI:** 10.1038/srep34829

**Published:** 2016-10-10

**Authors:** Kevin K. Ariën, Françoise Baleux, Delphine Desjardins, Françoise Porrot, Yves-Marie Coïc, Johan Michiels, Kawthar Bouchemal, David Bonnaffé, Timothée Bruel, Olivier Schwartz, Roger Le Grand, Guido Vanham, Nathalie Dereuddre-Bosquet, Hugues Lortat-Jacob

**Affiliations:** 1 Institute of Tropical Medicine, Virology Unit, Antwerp, Belgium; 2 Institut Pasteur, Unité de Chimie des Biomolécules, UMR CNRS 3523, 75015 Paris, France; 3 CEA, DRF/iMETI, IMVA-UMR1184, IDMIT infrastructure, Fontenay-aux-Roses, France; 4 Université Paris-Sud, UMR1184, Fontenay-aux-Roses, France; 5 Inserm, UMR1184, Center for Immunology of Viral Infections and Autoimmune Diseases (IMVA), Fontenay-aux-Roses, France; 6 Institut Pasteur, UMR CNRS 3569, Paris France; 7 Institut Galien Paris Sud, UMR CNRS 8612, Faculté de Pharmacie, Univ. Paris-Sud, Université Paris-Saclay, 92296 Chatenay-Malabry, France; 8 Institut de Chimie Moléculaire et des Matériaux d’Orsay, UMR 8182, LabEx LERMIT, Univ Paris Sud, CNRS, Université Paris-Saclay, Orsay, France; 9 University of Antwerp, Department of Biomedical Sciences, Belgium; 10 Institut de Biologie Structurale, UMR5075 CNRS, CEA, Université Grenoble-Alpes, 38027 Grenoble, France

## Abstract

The CD4 and the cryptic coreceptor binding sites of the HIV-1 envelope glycoprotein are key to viral attachment and entry. We developed new molecules comprising a CD4 mimetic peptide linked to anionic compounds (mCD4.1-HS_12_ and mCD4.1-PS1), that block the CD4-gp120 interaction and simultaneously induce the exposure of the cryptic coreceptor binding site, rendering it accessible to HS_12_- or PS1- mediated inhibition. Using a cynomolgus macaque model of vaginal challenge with SHIV162P3, we report that mCD4.1-PS1, formulated into a hydroxyethyl-cellulose gel provides 83% protection (5/6 animals). We next engineered the mCD4 moiety of the compound, giving rise to mCD4.2 and mCD4.3 that, when conjugated to PS1, inhibited cell-free and cell-associated HIV-1 with particularly low IC_50_, in the nM to pM range, including some viral strains that were resistant to the parent molecule mCD4.1. These chemically defined molecules, which target major sites of vulnerability of gp120, are stable for at least 48 hours in conditions replicating the vaginal milieu (37 °C, pH 4.5). They efficiently mimic several large gp120 ligands, including CD4, coreceptor or neutralizing antibodies, to which their efficacy compares very favorably, despite a molecular mass reduced to 5500 Da. Together, these results support the development of such molecules as potential microbicides.

Human Immunodeficiency Virus-1 (HIV-1), the virus that causes AIDS^1^, has infected over 60 million people worldwide. Although current treatments – mostly based on a combination of antiretroviral therapies - have highly improved patients’ outcomes, the virus continues to spread at a rate of ~1.5 million new infections per year. In that context, prevention of infection across the sexual mucosa, which is by far the predominant mode of transmission worldwide accounting for 90% of new infections, represents a valuable strategy to halt the pandemic^2^. Infection in the reproductive tract involves virus attachment to the mucosal epithelium, infection of subepithelial mononuclear cells and dissemination to the lymph node from where systemic infection develops^3^. Both initial attachment to mucosal cell surfaces and entry into permissive cells strongly depend on interactions between gp120, the glycoprotein which constitutes the outer part of HIV-1 envelope spikes (Env) and a variety of cell surface molecules^3^,^4^,^5^,^6^. These include lectins such as Dendritic Cell Specific ICAM-3-Grabbing Nonintegrin (DC-SIGN) or Mannose-Binding Lectin (MBL), some integrins, and glycans such as Galactoside Ceramide or Heparan Sulfate Proteoglycans (HSPGs), the latter being present at the surface of virtually all cell types^7^. Before encountering CD4-positive cells, the virus binding to these receptors can affect mucosal cells attachment and transport across epithelial layers, tropism and tissue invasion and bring Env into close proximity with CD4, thereby increasing the efficiency of infection. Any of these steps have thus been considered as logical targets for preventing transmission and a number of candidate microbicide molecules have been developed for intravaginal or intrarectal administration^8^,^9^.

Microbicides offer distinct advantages as their use should reduce side effects associated with systemic treatment and they could prevent the establishment of viral founder populations^2^,^3^,^9^,^10^,^11^. Up to now several molecules targeting the attachment and entry of HIV were tested in clinical trials, including surfactants, such as nonoxynol–9 and C31G that disrupt the viral lipid envelope and non-specific polyanions, such as carrageenan, cellulose sulfate, as well as a sulfonated naphthalene derivative (PRO2000), presumably mimicking heparan sulfate (HS) and blocking virus-cell interactions. Unfortunately, none of these compounds has demonstrated clear statistical evidence of protection in phase III clinical trials. Both surfactants and polyanions even showed increased risk of infection, presumably by causing mucosal epithelial damages allowing HIV-1 to enter epithelial tissues and/or promoting the formation of semen-derived amyloid fibrils which in turn enhances HIV-1 infection^8^,^12^,^13^,^14^,^15^. These disappointing results have underlined the need for the development of new agents targeting viral attachment in a more specific manner. In this regard, neutralizing antibodies, directed against the viral Env, and applied either locally^16^ or intravenously^17^ have been shown to protect macaques against a mucosal challenge with chimeric simian/human immunodeficiency virus (SHIV), displaying the Env of HIV-1. Encouraging results have also been obtained using nucleotide (NRTI) or non-nucleoside reverse transcriptase inhibitor (NNRTI) used in HIV/AIDS therapy which, incorporated in a vaginal gel formulation (Tenofovir) or administrated through a vaginal ring (Dapivirin), showed up to 40% reduction in HIV-1 acquisition^18^,^19^. Similarly, maraviroc, an entry inhibitor targeting the HIV-1 CCR5 coreceptor, formulated in aqueous gel demonstrated efficacy upon vaginal challenge in a rhesus macaques model^20^ and, administered through a vaginal ring, has recently completed a phase I study in humans^21^, validating the effectiveness of locally applied antiviral compounds.

In that context, we investigated here the anti-HIV activity of a recently developed molecule targeting gp120, using a model of vaginal infection in macaques. In contrast to the above mentioned molecules this compound combines both attachment and entry inhibition through a highly specific mechanism^22^,^23^ and inhibits gp120 binding to HS, CD4, and CCR5/CXCR4 coreceptors. Its design was based on previous studies showing that HS interacts with several regions of gp120, located close to each other, including the V2 and V3 loops and several residues within the co-receptor binding site^24^,^25^. These regions of gp120 are collectively involved in the conformational changes induced upon interaction with CD4, in particular the highly conserved four-stranded β sheet that becomes folded and/or exposed and which is critically involved in CCR5/CXCR4 recognition. Initial synthesis comprised a CD4 mimetic peptide (mCD4.1) linked to a chemically synthesized HS 12 mer. This molecule (mCD4.1-HS_12_) which showed IC_50_ in the 5–20 nM range against R5-, X4- and dual tropic HIV-1^22^ was further optimized by substituting the HS_12_ polyanion by a sulfopeptide giving rise to mCD4.1-PS1. As the parental compound, mCD4.1-PS1 binds gp120 through its mCD4 moiety and induces the structural modifications necessary to expose the coreceptor binding domain which, as a result, becomes available to be blocked by the PS1 moiety. This molecule neutralized R5- and X4- tropic HIV-1 of various clades with low nM IC_50_ in the absence of cellular toxicity^23^.

We have now assessed the ability of this compound at blocking infection in cynomolgus macaques, and report that it protected 83% (5/6) of the animals challenged vaginally with a single high dose of SHIV162P3. Following these *in vivo* results we further improved this compound, yielding mCD4.2-PS1 and mCD4.3-PS1 for which we found pM anti-viral activity against a range of HIV-1 strains and showed very efficient inhibition of HIV-1 cell-to-cell transmission, a major mechanism of viral spread and immune evasion.

## Results

### mCD4.1-PS1 inhibits several HIV-1 strains, including SHIV162P3, with better efficiency than mCD4.1-HS_12_

Previous work showed that mCD4.1-HS_12_ and mCD4.1-PS1 display nM IC_50_ towards a range of viral strains^22^,^23^. We first extended these observations, using the TZM-bl cell assay, with additional laboratory adapted- and clinical- isolates of HIV-1 (Table 1). We observed that mCD4.1 conjugates (either to HS_12_ or PS1) displayed more potent anti-viral activity than unconjugated mCD4.1 and that PS1 enhanced the activity of the molecule more importantly than HS_12_. Presumably, PS1 is a better mimic than HS_12_ of the N-terminal region of both CCR5 and CXCR4, which also features sulfotyrosine residues involved in gp120 recognition^26^. We noticed that the improvement in antiviral activity made by PS1 conjugation was more pronounced for R5- than X4- tropic viruses, the former being the major viruses transmitted between individuals^27^. In the case of SHIV162P3, the virus stock that will be used in the macaque challenge study, mCD4.1-HS_12_ and mCD4.1-PS1 displayed IC_50_ values of 706 and 36 nM, respectively (9.6 and 0.1 nM for the parental SF162 strain). Finally, mCD4.1-PS1 (Supplementary Fig. S1), which displays 6 sulfate groups versus 18 for mCD4.1-HS_12_ should be less prone to non-desired electrostatic-based interactions and, on these bases, was selected for *in vivo* evaluations.

### Stability and pharmacokinetics of mCD4.1-PS1 in the vaginal fluids

**Figure 1:**
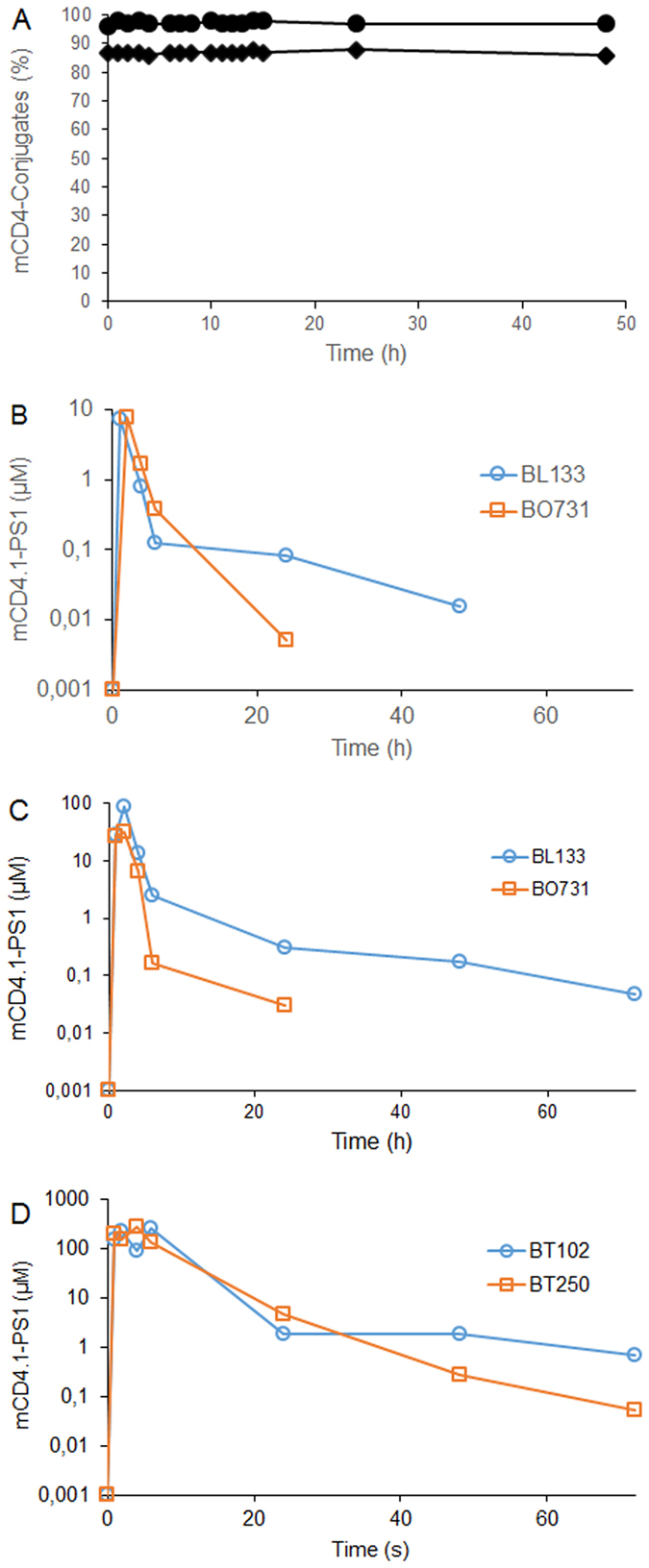
Stability and macaque pharmacokinetics. The mCD4.1-PS1 (diamonds; starting purity 87%) and the mCD4.1-HS_12_ (circles; starting purity 98%) dissolved at 1 mg/ml in 10 mM sodium citrate buffer pH 4 was incubated at 37 °C and regularly injected on a C18 RP-HPLC column over a 46 hours period. The integrity of the molecule was followed by monitoring its elution time using a 23–33% linear gradient of acetonitrile in 50 mM triethylamine acetate buffer and represented as mCD4.1-conjugates % content (**A**). Active mCD4.1-PS1 concentration measured in the vaginal fluid of two macaques at various times following vaginal administration of mCD4.1-PS1 formulated at 36 (**B**), 144 (**C**) or 600 (**D**) μM in a 1.5% HEC gel.

Before investigating the pharmacokinetic behavior of mCD4.1-PS1 in the vaginal mucosa, we first assessed its stability in conditions mimicking the human vaginal milieu. For that purpose, the molecule was solubilized in a pH 4.5 citrate buffer and incubated at 37 °C. HPLC analysis, performed at various time points, did not show modifications for up to 48 h of both mCD4.1-PS1 and mCD4.1-HS_12_ (Fig. 1A). Formulated in HEC gel and stored at −20 °C, mCD4.1-PS1 was also stable for one year (data not shown). Next, the molecule was prepared at 36, 144 or 600 μM in 1.5% HEC hydrogel in 5 mM citrate buffer pH 4.5, 0.1% sorbic acid and 2.5% glycerol. These preparations (2 ml of gel) were then administrated in the vaginal cavity of anaesthetized and Depo-Provera pre-treated female cynomolgus macaques (two animals per condition), after which vaginal fluid sampling was performed at baseline, 1, 2, 4, 6, 24, 48 and 72 hours. To quantify the mCD4.1-PS1 activity in each sample we evaluated vaginal fluid dilutions for their inhibitory potency against the HIV-1 IIIB strain. This viral strain features a very high sensitivity to mCD4.1-PS1 (IC_50_ = 0.013 nM) compared to the other virus investigated (Table 1) and thus constitutes an excellent reporter of the compound activity. Knowing the IC_50_ of mCD4.1-PS1 against this particular strain, the amount of active mCD4.1-PS1 remaining in the cervico-vaginal fluids (CVL) could be calculated as a function of time. An initial low dose of 36 μM gave rise to measurable anti HIV activity, corresponding to approx. 8 μM during the two first hours (only in one animal), dropping to below 1 μM afterwards. When applied at 144 μM, the mCD4.1-PS1 activity in CVL remained above 25 μM after 2 h and then declined rapidly. Finally, applied at a dose of 600 μM, mCD4.1-PS1 remained at a level above 150 μM (i.e. 4000-fold the IC_50_ against the SHIV162P3) during the first 6 h, then dropped to approx. 3.5 μM at 24 h (Fig. 1B–D).

### *In vivo* challenge of macaques

**Figure 2:**
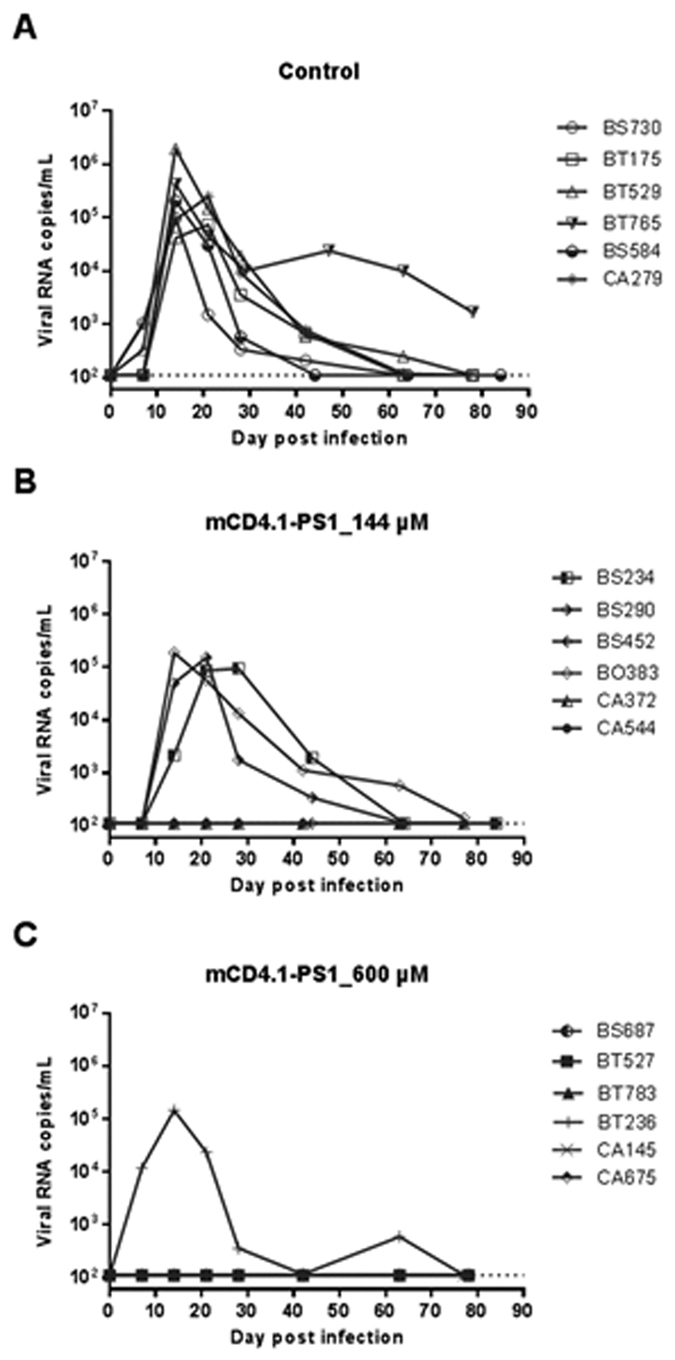
Efficacy of mCD4.1-PS1 formulated in HEC gel to prevent vaginal transmission of SHIV162P3 in macaques. Plasma viral load in SHIV162P3 challenged macaques treated with placebo gel (**A**) or gel containing mCD4.1-PS1 at 144 μM (**B**) or 600 μM (**C**). Each gel formulation was applied into the vaginal vault of six naïve female cynomolgus macaques 1 hour before the challenge performed with ~10 AID_50_ of SHIV162P3 inoculated in 50% human seminal plasma. Plasma viremia were measured regularly for 11 weeks by quantitative RT-PCR. The dotted line on the y-axis represents the 111 RNA copies/mL quantification limit.

For the *in vivo* challenge, 3 groups of 6 female cynomolgus macaques were treated with Depo-Provera one month before challenge to synchronize the menstrual cycle and thin the vaginal epithelium which increases the susceptibility to infection after a single exposure^28^. Animals homozygous for H6 haplotype of MHC-I reported to be less susceptible to progression to disease after SIV infection^29^ were excluded from the study. Animals received 2 ml of HEC gel containing no, 144 or 600 μM of mCD4.1-PS1, applied into the vaginal vault one hour before the challenge. The 36 μM formulation was not investigated, as several previous studies showed that *in vivo* efficacy requires concentration several orders of magnitude higher than the *in vitro* IC_50_^16^,^30^,^31^,^32^,^33^,^34^. All 6 control animals that received the placebo gel became infected (Fig. 2A). The mCD4.1-PS1 HEC gel at 144 μM provided partial protection with 3 out of 6 (50%) animals remaining SHIV RNA negative and seronegative during the course of the study (Fig. 2B). In contrast, only 1 out of 6 animals receiving the mCD4.1-PS1 HEC gel at the concentration of 600 μM became infected (Fig. 2C) resulting in a significant difference when compared to the control group (83% of protection: Fisher exact’s test, p = 0.0152). The five protected animals treated with the mCD4.1-PS1 HEC gel at 600 μM remained seronegative throughout the duration of the study and we confirmed that none of the protected animals had a detectable SHIV DNA in lymph nodes collected at week 9 after challenge (data not shown). For the four animals infected in the two mCD4.1-PS1 HEC gel groups, SHIV viruses isolated from plasma were sequenced to document any evidence of resistance. No reported resistance mutations were detected and none of the isolates had the key mutation in amino acid position 375 of gp120 that was previously shown to confer resistance to mCD4 *in vitro*^35^.

### mCD4.1-PS1 optimization

**Figure 3:**
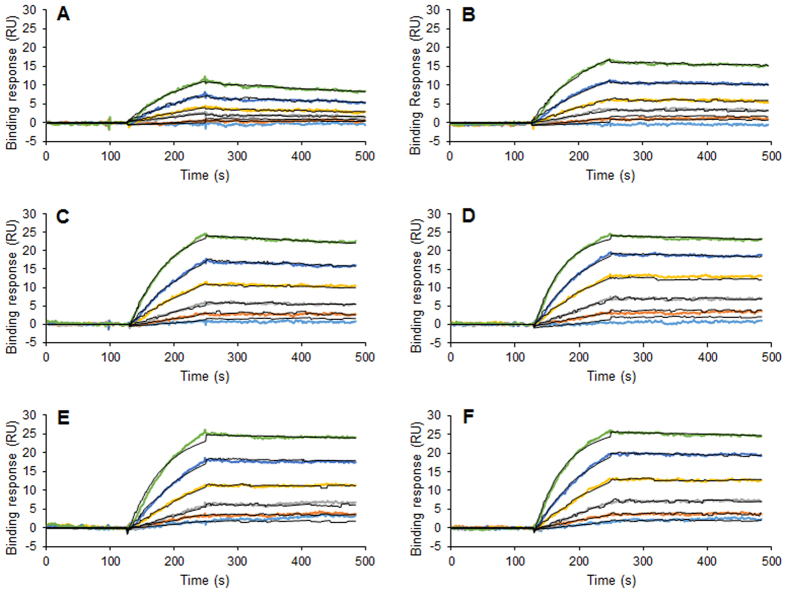
Kinetic analysis of the binding of mCD4-PS1 conjugates to gp120. Surface plasmon resonance sensorgrams measured when mCD4.1-PS1 (**A,B**), mCD4.2-PS1 (**C,D**) or mCD4.3-PS1 (**E,F**) at 5, 2.5, 1.25, 0.62, 0.31 and 0.15 nM (from top to bottom), were injected on HIV-1 MN (**A,C,E**) or YU2 (**B,D,F**) gp120. The binding response in RU was recorded as a function of time (colored curves), and fitted to a Langmuir binding model (black curves).

Having optimized the anionic moiety of the molecule, by conjugating mCD4.1 to PS1 rather than to HS_12_ and having established that the resulting molecule efficiently prevents acquisition of infection, we made use of a recently improved CD4 mimetic M48U1^36^ to further enhance the activity of this family of compounds. This mimetic features a solvent accessible Lys residue at position 11 that could have been used for conjugation purpose. However, structural data analysis of core gp120 in complex with such class of CD4 mimetic showed that this position is too far from the gp120 coreceptor binding domain to be targeted by PS1^22^. Thus, we engineered a mutation at position 5 (F5K) which is also solvent accessible and only 10 Å away from the co-receptor binding domain to allow PS1 coupling as well as K11S or K11R mutations to avoid multiple derivatization points (Supplementary Fig. S1 and Table S1). These two new peptides, mCD4.2 and mCD4.3 respectively, where then conjugated to PS1, giving rise to mCD4.2-PS1 and mCD4.3-PS1 (Supplementary information method). To quantify the affinity of these molecules for R5- or X4- derived gp120, we performed direct binding analyses in which series of concentrations of the conjugates were injected over X4- or R5- gp120 immobilized on a sensorchip. Surface Plasmon Resonance (SPR) monitoring was used to measure changes in refractive index caused by the interaction and the data were fitted to a Langmuir binding model. Results are shown in Fig. 3 and indicate that mCD4.2-PS1 and mCD4.3-PS1 display very high affinity for their targets with K_D_ = 0.098 and 0.056 nM for HIV-1 MN gp120 and 0.032 and 0.038 nM for HIV-1 YU2 gp120, used as models of X4- and R5- Env respectively. By comparison, the initial mCD4.1-PS1 binds MN- and YU2- gp120 with K_D_ of 0.5 and 3.26 nM respectively (Fig. 3), demonstrating from a biochemical point of view the improvement achieved when PS1 was conjugated to mCD4.2 and mCD4.3.

### mCD4.2-PS1 and mCD4.3-PS1 display enhanced anti HIV-1 activity

The antiviral activity of mCD4.2-PS1 and mCD4.3-PS1 was determined against a number of HIV strains from different subtypes, in direct comparison with the corresponding unconjugated mCD4.2 and mCD4.3, as well as the gp120 CD4 binding site-neutralizing mAbs VRC01 and b12. The NNRTI dapivirine was also used as a reference with a different mode of action and more conserved target. The CD4 mimetic mCD4.2 and mCD4.3 were about 2 logs more active than the parent mCD4.1 (average decrease in IC_50_ is 80 and 115 times for mCD4.2 and mCD4.3, respectively). Addition of the PS1 to mCD4.2 and mCD4.3 further increased the potency of the compounds against all strains tested, with factors ranging between 2- and 270-fold giving rise to compounds with low nM or pM activity (Table 2). As these compounds remained devoid of any cellular cytotoxicity they feature a remarkably high safety window (CC_50_/IC_50_). As compared to the well-characterized CD4 binding site-specific mAb VRC01 and b12, mCD4.2-PS1 and mCD4.3-PS1 were also more active against most viruses tested and appeared to be as active as 3BNC117, one of the most potent anti-CD4 binding site antibodies that have been shown to neutralize 195 out of 237 HIV-1 strains with an average IC_50_ of 0.5 nM^37^. As expected from our previous work^38^,^39^ the NNRTI dapivirine was active at 1–2 nM, except against SHIV162P3, since this virus contains the SIV RT, which is naturally resistant to all NNRTI. Importantly, whereas the subtype C viruses VI829, p246F10 and pZM247Fv2 appeared all resistant to the parent compound mCD4.1 (IC_50_ > 10,000 nM – see Table 1), they were highly sensitive to the most powerful compound mCD4.3-PS1 (IC_50_ = 3.6, 33, and 192 nM respectively – see Table 2). Of note, we tested five transmitted/founder (T/F) viruses which were all inhibited with nanomolar concentrations of the PS1 conjugates of either mCD4.2 or mCD4.3. Remarkably also, out of the two CRF01_AE viruses tested, VI1888 was highly resistant to all mCD4 (IC_50_ >10,000 nM), but could be inhibited by approx. 1 μM concentration of the mCD4 conjugated to PS1. In contrast, the Ca10-3 was sensitive to all compounds. Previous observations^35^,^40^ indicated that the Ser residue at amino acid position 375 in gp120 is highly conserved across HIV-1 subtypes. Interestingly, most CRF01_AE viruses contain a His at that position, likely explaining their resistance to CD4 miniproteins^35^, except for virus Ca10-3, which has a Ser 375.

### mCD4-PS1 inhibits HIV-1 cell-to-cell transmission

**Figure 4:**
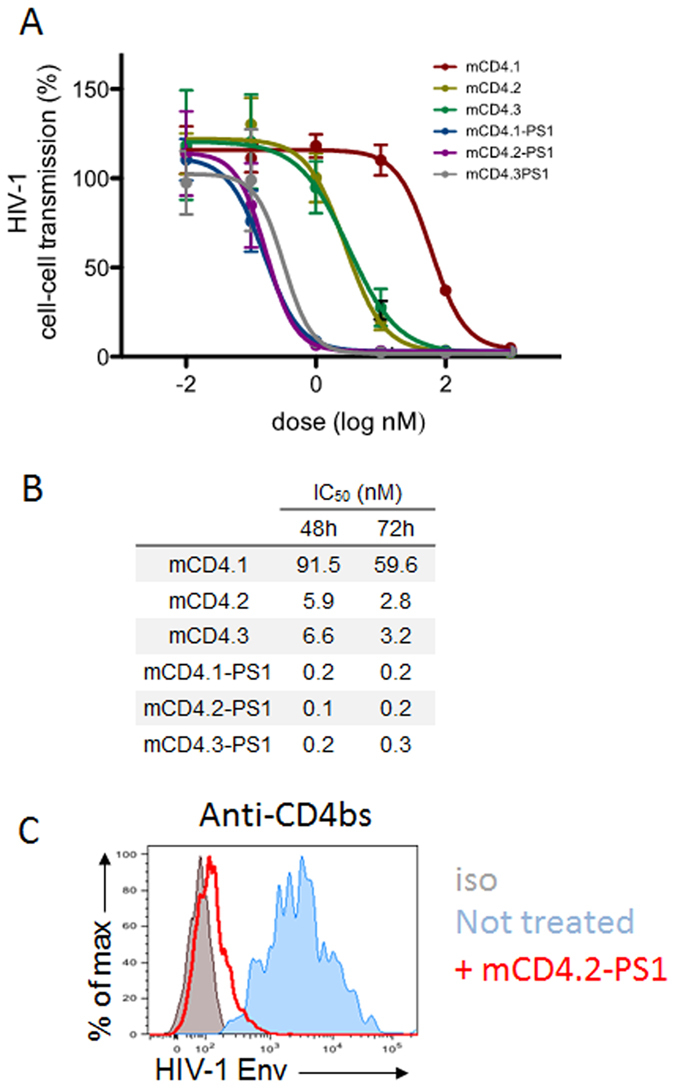
mCD4-conjugates inhibit cell-cell transmission and bind cell-associated HIV-1 Env. (**A**) HIV cell-cell transmission. Primary CD4 T-cells were infected with the X4-tropic HIV-1 strain NL4.3 and co-cultivated with dye-labeled autologous uninfected CD4 T-cells, in the presence of indicated amounts of inhibitors. After 72 h of culture, the infection of target cells (fraction of Gag+ cells) was measured by flow cytometry. Data are mean ± SEM of three independent experiments, with 100% corresponding to the levels of Gag+ targets in the absence of inhibitor. (**B**) IC_50_ (in nM) of the inhibitors calculated with the dose-response, at two different time points (48 and 72 h). (**C**) HIV-1 Env surface levels. CEM-NKR cells infected with HIV-1 NL4.3 were exposed to mCD4.2-PS1 (1 μM) for 10 min at 37 °C and then stained with an antibody targeting the HIV-1 Env CD4 binding site (3BNC117). Binding was examined by flow cytometry. One representative experiment out of three is shown.

Although cell-free HIV is infectious, HIV-1 spreads more efficiently and rapidly through direct contact between cells which represents a major mechanism of viral spread and immune evasion *in vivo*^41^,^42^,^43^. We thus evaluated the ability of the mCD4 constructs to block this mode of viral spread. Primary CD4^+^ T cells infected with NL4.3 HIV-1 strains were incubated with mCD4 or mCD4-conjugates, before co-culture with autologous target cells labeled with FarRed. Infection of target cells was measured by Gag expression after 48–72 h (Fig. 4). The contribution of cell-free virus to infection was negligible, since Gag expression by the recipient cells was abrogated by separation of donors and targets in a transwell chamber, or when cultures were gently shaken to avoid prolonged contacts^43^. Moreover, Gag expression in target cells was due to *de novo* synthesis, since it was substantially reduced in the presence of nevirapine or with the broadly neutralizing antibody (bNab) targeting the CD4 binding site on Env (NIH45–46 bNAb, data not shown and^44^). The mCD4.1, 2 and 3 constructs inhibited HIV-1 cell-cell transmission at a high concentration: 100–1000 nM (Fig. 4A). Strikingly, the mCD4.1,2 or 3-PS1 constructs were much more active and blocked by more than 95% HIV cell-cell spread at 1 nM, with IC_50_ = 0.1–0.3 nM (Fig. 4A,B). To describe further the mechanism of action of the mCD4-PS1 constructs, we incubated HIV-1 infected cells with mCD4.2-PS1, chosen as a representative compound of the mCD4-PS series, for a short period (10 min at 37 °C). Then we measured the levels of Env epitopes at the cell surface using the 3BNC117 bNAb that targets the CD4bs. We observed that binding of this antibody was strongly inhibited (Fig. 4C), therefore, mCD4.2-PS1 efficiently binds the CD4 binding site of Env glycoproteins present at the surface of infected cells. Altogether, our results indicate that mCD4-PS1 constructs act at low concentrations to potently inhibit HIV-1 cell-to-cell transmission.

## Discussion

Despite continuing advances in the treatment and prevention of HIV-1 infection, the virus continues to spread, both in developed and developing countries where it represents an important and unsolved problem. Thus, although the introduction of combined antiretroviral drugs has greatly improved the patients’ survival and quality of life, alternative approaches to curb the epidemic are being pursued. Prevention, by entry- or reverse transcriptase inhibitors, is regarded as a valuable strategy as it blocks HIV-1 at a step prior to viral DNA integration into the host genome. Such approach includes the use of antiretroviral compounds that can be applied either systemically as oral preexposure prophylaxis (oral PrEP) or locally at the mucosal site of virus entry. Vaginal or rectal microbicide formulations, in particular, represent a potentially efficient way to prevent the initial stages of HIV-1 entry and halt the viral spread that mostly occurs through sexual transmission^3^,^5^,^8^,^9^,^10^,^11^.

In that context, we used a cynomolgus macaque model of vaginal transmission to test whether a new “attachment-entry” inhibitor, mCD4.1-PS1, formulated into a HEC gel and locally applied, would prevent infection. This compound is a “multispecific” agent that binds several regions of vulnerability of the virus Env. It was designed to circumvent the conformational masking mechanisms that protect the highly conserved bridging sheet of gp120, a critical component of the coreceptor binding site: while the mCD4 moiety blocks the CD4-gp120 interaction, it induces the exposure of the cryptic coreceptor binding site and renders it available for interaction with the sulfotyrosine containing peptide PS1. This peptide, in addition, mimics a number of gp120 ligands, including HS^23^ and several “CD4-induced antibodies” that also comprise sulfotyrosines^45^. It thus targets the coreceptor binding sites and epitopes of the V2 and V3 loops.

PK studies showed that of the three mCD4.1-PS1 dosages (i.e. 36, 144 or 600 μM) in HEC gel, a formulation widely used for vaginal delivery^46^ only the highest dose yielded an active concentration of the compounds within the vaginal vault above 150 μM (thus approx. 4000 fold the IC_50_ of this compound against the SHIV162P3) during the first 6 h, while the lowest one only gave rise to low μM activity. In similar studies, where gel formulated molecules (including Rantes, CD4-mimetic, neutralizing antibodies, cyanovirin or maraviroc) were vaginally administered, the viral challenge was performed 30 to 60 minutes following compound application, suggesting residence times comparable to the ones we report for mCD4.1-PS1. When pharmacokinetic studies were conducted (*i.e.* with neutralizing antibodies, CD4 mimetic or maraviroc) the data indicate that they are also within a time range of a few hours^16^,^20^,^30^,^32^,^47^.

Vaginal challenge resulted in infection of all the control animals, while in the animals treated with 144 and 600 μM of mCD4.1-PS1, 50% (3/6) and 83% (5/6) were fully protected respectively. A significant difference was observed between the animals treated with the mCD4.1-PS1 at 600 μM and animals treated with the placebo gel (Fisher’s exact test, p = 0.0152). The study was powered to detect over 67% efficacy. In infected animals, viral load could not be distinguished from controls animals as we reported previously with prevention obtained with gels containing monoclonal neutralizing antibodies^16^. Co-factors like MHC haplotype or menstrual cycle could not explain infection since animals with genetic background for infection susceptibility had been excluded and females were all treated with Depo-provera.

Consistently with the present results, a number of studies, employing similar models, with either biologics or small drugs showed that compounds inhibiting viral entry or attachment with nM activity *in vitro* provided protection *in vivo* only when applied at mM concentration. For example, early studies with the RANTES derivative PSC-RANTES, a chemokine binding to CCR5 and inhibiting SHIV SF162 replication in rhesus PBMC with subnanomolar IC_50_, required to be administered at 0.1, 0.3 or 1 mM to provide 60, 80 and 100% of protection, respectively^31^. Similarly, HEC gel formulated cyanovirin was shown to protect 5 out of 6 animals (83%) when applied at 0.5 to 2 mM^32^ and a combination of three neutralizing antibodies at 20 mg/ml each was required to achieve 70% protection^16^. Finally, a miniCD4 derivative that displayed 25 nM IC_50_ against the SHIV162P3 also protected 83% of the animals with 1 mM formulation^30^. The high difference observed between the *in vitro* and *in vivo* efficacy is not fully understood, but amongst the possible reasons are the interference with a complex local environment comprising many proteins, the endogenous microbiota and the microbial metabolites^5^ and/or the poor stability of the compounds. It should be pointed out, nevertheless, that the same route of delivery used for small drugs, that by nature are more stable, gave very similar results. For example, 6 of 7 macaques remained uninfected when receiving 6 mM of maraviroc intravaginally, while 500 μM gave rise to only 50% protection^33^. Similarly, the compounds BMS-378806 and CMPD167 that block SHIV162P3 with respectively 5.5 and 0.22 nM IC_50_ in TZM-bl cells, gave rise to 75% and 80% protection when administered at 5.5 and 5 mM respectively^34^. It is possible that escape was due to imperfect gel delivery or a local inflammatory response that may interfere with infection, for example by recruiting susceptible cells. Finally, as suggested by the dose/response study conducted here it is also possible that the dose applied was not sufficient to achieve 100% of protection, which, by extrapolation could be obtained with a 0.8–1 mM dose.

Compared to the above mentioned compounds, the mCD4.1-PS1 we investigated here has a number of particularities and advantages, in addition to its high efficacy, evidenced by 83% of protection when applied at 0.6 mM. A first one is its multispecific nature. It targets both the CD4 and the coreceptor binding sites, thus blocking the interaction with CD4, CCR5 and CXCR4. The advantage of multispecificity has been recently illustrated in a study showing that 25 mAbs isolated from a patient who had suppressed HIV-1 infection for more than 25 years without any treatment, have only limited potency and breadth in neutralization at the individual level, while the polyclonal combination, present *in vivo*, was apparently effective. Interestingly, the antibodies from this patient with non-progressive disease were shown to target the V3 loop, the CD4 binding site and the CD4 induced epitope^48^. All these sites are also targeted by the mCD4.1-PS1 molecule. The sulfopeptide moiety is also mimicking HS, a ligand of gp120 that was previously shown to interact with the V2 and V3 loops^24^,^25^.

Whereas the gp120-HS interaction has been well described both *in vitro* and *in cellulo*^7^, the role it could play *in vivo* is not completely clear. It has been reported nevertheless that HIV-1 transmission is reduced in children developing kwashiorkor compared to those suffering from marasmus, two forms of severe malnutrition, the former being characterized by a markedly reduced production of HS featuring a low sulfation profile, while the expression and the nature of HS were similar between marasmic and well-nourished children^49^. HIV-1 entry across epithelial or endothelial barriers as well as transplacental infection might thus be HS dependent, and it will be of interest to investigate whether the CD4-conjugate molecules could interfere with these processes. Interestingly, it has also been reported that spermatozoa can capture HIV-1 in an HS dependent manner, and efficiently transfer virions to dendritic cells, macrophages and T-cells to which access is made possible through mucosal microabrasion^50^. It can be hypothesized that this process of trans-infection is also susceptible to inhibition by the mCD4-sulfopeptide compounds.

Another line of interest of this class of molecules, is that the efficacy of its two moieties can be improved independently. It can thus be considered as a flexible and multifunctional platform which can give rise to modular developments. We already demonstrated that mCD4.1-PS1 displayed enhanced anti HIV-1 activity compared to mCD4.1-HS_12_^23^. After having ameliorated the anionic domain of the molecule, we further improved the mCD4 moiety. Two new molecules i.e. mCD4.2 and mCD4.3 were prepared for that purpose and conjugated to PS1, giving rise to mCD4.2-PS1 and mCD4.3-PS1. SPR-based binding experiments indicated that these new conjugates displayed very high affinity for both HIV-1 MN and YU2 gp120 with K_D_ in the pM range. Using the TZM-bl assay we found that although mCD4.2 and mCD4.3 already feature 100-fold increased anti-viral activity (as compared to mCD4.1) their conjugation to PS1 further strongly increased their efficacy, regardless of the viral strain investigated, including both subtype B and C transmitted/founder viruses. Being obtained by total chemical synthesis, these molecules are fully defined and can thus be specifically designed and tailored to accommodate new modalities. It would be conceivable, for example, to link a further peptide targeting the Env gp41 subunit.

To better analyze the potential usefulness of this new series of molecules, we measured their ability to inhibit transmission of cell-associated viruses, which, in addition to cell-free virions are present in semen and genital secretion. This mode of transmission is largely mediated by the virological synapse, where virions accumulate at the interface between infected and target cells^42^,^51^. It is known that HIV-1 viruses spread more efficiently through this cell-to-cell mechanism. It is thus likely that this form of transmission, which in addition is less susceptible to inhibition by neutralizing antibodies^44^,^52^ or antiretroviral drugs^53^ than cell-free virus, represent an important fraction of viral spread and immune evasion^41^,^42^,^43^. In this context, we report here that the mCD4-conjugates also behave as very efficient inhibitors of cell-associated viruses, 95% of HIV-1 cell-to-cell spread inhibition being achieved at concentration as low as 1 nM. Consistent with the biochemical and cell-free virus infection, the addition of the sulfopeptide to the mCD4 enhanced the efficacy of the molecules for inhibition of HIV-1 cell-to-cell transmission by several orders of magnitude. We previously analyzed a panel of bNabs, for their ability to neutralize HIV-1 cell-to-cell transmission and reported an average IC_50_ of 5 nM^44^. Compared to these antibodies, the best CD4-PS1 conjugates (i.e. mCD4.2-PS1 and mCD4.3-PS1) are much more potent, with IC_50_ of 0.1–0.2 nM. Their reduced molecular masses (5500 Da), compared to that of antibodies, may facilitate their access and accumulation at the virological synapses. Such low IC_50_ suggests that these improved compounds could be active *in vivo* using much lower concentrations than the previous generations of entry inhibitors (i.e. mCD4.1-HS_12_ and mCD4.1-PS1). Considering the SHIV162P3, for example, a 20-fold enhancement was observed between mCD4.1-PS1 and the new molecules, suggesting that protection could achieve at much lower concentrations than the 600 μM dosage used. Combined with the absence of cytotoxicity at high concentration, this study validates the interest of the mCD4-PS molecules as anti HIV-1 agents. In particular, their multifunctional mode of action, their high activity, both against cell-free and cell-associated viruses at sub-nM IC_50_ support the exploration of novel and recently developed routes of administration that could maximize the activity, safety, acceptability and adherence.

It has been recently shown that the *i.v.* administration of a cocktail of HIV-1 specific and broadly neutralizing Abs, comprising PGT121, 3BNC117 and b12, resulted in a rapid decline of plasma viremia to undetectable levels in rhesus monkeys chronically infected with the SHIV-SF162P3^54^. *I.v.* injection of 3BNC117, one of the most potent anti-CD4 binding site neutralizing antibody reported to date, with a plasma half-live of 3.3 days when injected at 5 mg/kg also prevents the acquisition of SHIV_AD8-EO_ infection following rectal challenge^55^ and in infected humans, where the half-life was 9 days, a 30 mg/kg injection reduced the viral load by 0.8 to 2.5 log_10_^37^. It thus would be also of interest to know if systemic treatment with the mCD4-conjugate would have the same effect. Such development raises the question of the potential immunogenicity of this class of compound, a point of concern that is subject of future studies. It is worth noting, however, that immunization with multiple high doses of a chimeric antigen comprising gp120 linked to CD4 sequences did not elicit detectable autoantibody reactivity with cell surface CD4, despite a robust response to the antigen^56^.

## Methods

### Ethics statement

Adult cynomolgus macaques (*Macaca fascicularis*) were imported from Mauritius and housed in the facilities of the Commissariat à l’Energie Atomique et aux Energies Alternatives (CEA). Non-human primates (NHP) are used at the CEA in accordance with French national regulations and under national veterinary inspectors (CEA Permit Number B 92-032-02). The CEA complies with the Public Health Service Policy on Humane Care and Use of Laboratory Animals of the Office for Laboratory Animal Welfare (OLAW, USA) under OLAW Assurance number #A5826-01. All experimental procedures were conducted according to the newly published European directive (2010/63, recommendation N°9). The protocols employed were approved under statement number 14-060 by the ethics committee “Comité d’Ethique en Expérimentation Animale du CEA” registered with the French Ministry of Research under N°44. The animals were used under the supervision of the veterinarians in charge of the animal facility. Handling procedures were conducted after animal sedation with ketamine chlorhydrate (Rhone-Merieux, Lyon, France; 10 mg/kg body weight).

### Sulfopeptide, CD4 mimetics and conjugates synthesis

mCD4.1-HS_12_ and mCD4.1-PS1 were produced as previously described^22^,^23^ and mCD4.2-PS1 and mCD4.3-PS1 were prepared using the same protocol. Briefly, after mCD4 peptide synthesis, a maleïmide group was selectively introduced on the NH_2_ε LYS_5_ side chain. In parallel, a thioacetyl group was introduced at the N-terminus of PS1 sulfotyrosines peptide. Hydroxylamine treatment generated a free thiol onto PS1 that allowed the covalent linkage to maleïmide activated mCD4 peptides to yield the desired conjugates. Peptides were prepared by solid-phase synthesis using Fmoc chemistry. All compounds were purified by RP-HPLC and characterized as described in the supplementary information.

### Peptide formulations

Hydrogels containing mCD4.1-PS1 were prepared by successively mixing the peptides (so as to have a final concentration of 36, 144 or 600 μM), hydroxyethylcellulose (HEC) gelling polymer (1.5 wt%), citrate buffer, sorbic acid (0.1 wt%) and glycerol (final concentration 2.5 wt%). Placebo gels, in which the peptide was omitted, were prepared similarly. The formulations were mixed until complete dissolution of HEC. The viscosity of the placebo hydrogel was 3.2 Pa.s and 1.9 Pa.s at 25 °C and 37 °C, respectively, the osmolarity was 287 mOsm/kg and the final pH 4.5.

### Stability studies

The mCD4.1-PS1 and mCD4.1-HS_12_ were dissolved at 1 mg/ml in 10 mM sodium citrate buffer pH 4.5 to mimic the human vaginal condition, and incubated at 37 °C. Compound stability was evaluated by regularly injecting 2 μl aliquots on a C18 RP-HPLC (Waters Symmetry column, 3.5 μm, 300 Å, 2.1 × 100 mm) over a 46 hours period. The integrity of the molecule was followed by monitoring its elution time and % content using a 23–33% linear gradient of acetonitrile in 50 mM triethylamine acetate buffer over 20 minutes at a 0.35 ml/min flow rate (detection 230 nm). The staring purity of the compounds, as measured by HPLC, were was 87 and 98% respectively.

### Pharmacokinetics in NHP

A PK study of the mCD4.1-PS1 was conducted in naïve female cynomolgus macaques pre-treated intramuscularly with 30 mg medroxyprogesterone acetate (Depo-Provera, Pfizer) 4–5 weeks before the gel application. Two ml of HEC gels containing mCD4.1-PS1 at 36, 144 or 600 μM, were applied into the vaginal cavity with a French catheter connected to ready-to-use syringe (two females per group). Vaginal fluids were collected before and at various time points after gel administration, by placing pre-weighted Weck-Cel sponges (Beaver Visitec International) into the vagina. Upon removal, sponges were reweighed to calculate the collected vaginal fluid weight and sponge contents were eluted in PBS containing 0.25 M NaCl and protease inhibitors (Calbiochem). Eluates were stored at −80 °C prior to analysis. The mCD4.1-PS1 activity remaining in these samples, diluted to at least 3% to eliminate any cytotoxicity, was measured in TZM-bl cells using the HIV-1 IIIB strain that constitutes an excellent reporter of the compound activity. Animals BL133 and BO731 included in the PK study at 36 μM were re included in the PK study at 144 μM. In that case, a one week wash out period was introduced between the two studies, and sampling before compound re-application demonstrated the absence of any remaining activity.

### Challenge of NHP with SHIV162P3

SHIV162P3 was obtained from the NIH AIDS Research and Reference Reagent Program^57^,^58^ and mixed with 50% human seminal plasma^59^. This mixture was used at 10 animal infectious doses 50% (AID_50_) to vaginally infect 18 naïve female cynomolgus macaques, which had been pre-treated with Depo-Provera 4–5 weeks before the challenge. One hour before challenge, six macaques received an intravaginal dose of 2 mL of mCD4.1-PS1 containing HEC gel at 144 μM. Six other macaques received the same dose of HEC gel containing 600 μM of mCD4.1-PS1 and the other six macaques were dosed with HEC gel without mCD4.1-PS1 (placebo group). Blood was collected at different time points after challenge to measure plasma viremia by a quantitative RT-PCR assay using primers amplifying the *gag* regions of SIVmac251. The detection limit of this assay is 60 RNA copies/mL and the quantification limit is 111 RNA copies/mL. For animals with undetectable viremia, lymph nodes were sampled on week 9 after challenge to measure the SHIV DNA copy numbers by quantitative PCR. Detection limit for this assay is 10 copies per million of cells^60^. Anti-SHIV binding antibodies were measured in serum using standard commercial ELISA for the detection of HIV-1 and HIV-2 antibodies (Genscreen, Bio-Rad). Animals were considered negative if tested negative for plasma viremia and antibodies over the course of the study (11 weeks of follow-up after challenge) and negative for proviral DNA in lymph nodes.

### Antiviral assay

The IC_50_ of the various mCD4-conjugates were measured in TZM-bl cells (NIH AIDS Reagent Program, Germantown, MD 20874, US). This cell line expresses high densities of CD4, CCR5 and CXCR4. It contains a luciferase reporter gene under control of HIV LTR, which will be transcribed and translated into luciferase protein, if the cells get infected with HIV and start producing Tat. Briefly, 50 μl of TZM-bl cells (1 × 10^5 ^ cells/mL) supplemented with 30 μg/mL DEAE dextran were incubated with a 50 μl of a 10-fold serial dilution of the compounds under investigation in 96-well flat bottom plates, prior to inoculation with 50 μl of various viruses (see Tables & and 2; 50, 100 or 200 TCID50 was used depending on the virus). As a positive control, 50 μl of compound-free medium was used and as a negative control, 100 μl of medium was added to 100 μl of TZM-bl cells. All conditions were done in triplicates. The plates were incubated for 48 hours (37 °C, 5% CO_2_). Subsequently, 120 μL of supernatants were removed, 75 μl of the luciferase substrate Steadylite (Perkin Elmer, Life Sciences, Zaventem, Belgium) were added to the wells and the plates were incubated at room temperature on an orbital shaker for 10 minutes. Next, the luciferase activity was measured using a TriStar LB941 luminometer (Berthold Technologies GmbH & Co. KG., Bad Wildbad, Germany) and expressed in relative light units (RLU) as a percentage of positive control wells. IC_50_ were calculated in GraphPad Prism 5.03 using non-linear regression (GraphPad Software, San Diego, CA, USA).

### Cytotoxicity

The water soluble tetrazolium-1 (WST-1) cell proliferation assay is based on the cleavage of the tetrazolium salt WST-1 to a formazan dye by a complex cellular mechanism. Because this bioreduction is dependent on the glycolytic production of NAD(P)H in viable cells, the amount of formazan dye formed is correlates directly to the number of viable cells in a culture. Quantification is done by measuring absorbance at 450 nm in a multiwell plate reader. TZMbl cells (10^4^ cells/well) were plated in a 96-well plate and a serial dilution of compound was added. 48 h later, cell proliferation reagent was added and cell viability was measured compared to untreated control cultures. Cell viability was plotted against the compound concentration and non-linear regression analysis was performed to calculate the 50% cytotoxic concentration (CC_50_).

### Compound dosing in cervico-vaginal lavage fluids (CVL)

CVL were cleared from any cell-debris by centrifugation at 1,500 rpm during 10 minutes, prior to freezing at −80 °C. After thawing, TMZbl cells (10^4 ^ cells/well) were incubated with a serial 10-fold dilution (starting from 3% down to 0.00003%) of each CVL for 30 minutes. Subsequently, 50 TCID50 of IIIB virus was added to the cells. All conditions were done in triplicates. The plates were incubated for 48 hours (37 °C, 5% CO_2_). Subsequently, 120 μL of supernatants were removed, 75 μl of the luciferase substrate Steadylite (Perkin Elmer, Life Sciences, Zaventem, Belgium) were added to the wells and the plates were incubated at room temperature on an orbital shaker for 10 minutes. Next, the luciferase activity was measured using a TriStar LB941 luminometer (Berthold Technologies GmbH & Co. KG., Bad Wildbad, Germany) and expressed as relative light units (RLU) and expressed as a percentage of that in positive control wells. IC_50_ were calculated in GraphPad Prism 5.03 using non-linear regression (GraphPad Software, San Diego, CA, USA) and used to estimate the compound concentrations in CVL.

### Surface Plasmon Resonance based binding assay

The interactions between gp120 and its ligands were analyzed by Surface Plasmon Resonance (SPR) based methods. For that purpose, N-ethyl-N’-(diethylaminopropyl)-carbodiimide (EDC)/N-hydroxy-succimide (NHS) activated CM4 sensorchips were functionalized with either MN or YU2 gp120 as described^23^ and the molecules under investigation were injected over the different surfaces. Binding responses were recorded as a function of time and analyzed by fitting both association and dissociation phases for several concentrations, using the Biaevaluation 3.1 software.

### Viral cell-to-cell transmission assay

Primary CD4^+^ T cells were purified from human peripheral blood by positive selection (Miltenyi). About 98% of cells were CD4^+^CD3^+^. For activation, primary T cells were treated with PHA (1 μg/ml) for 24 h and then cultured with IL-2 (50 IU/ml) for 3–5 days before use. Primary cells were infected with HIV-1 NL4-3 and NLAD8 as described^44^,^61^. Donor cells were used a few days later, when about 20% of the cells were Gag^+^. Target cells were labeled with FarRed (2.5 μM; Molecular Probes). Donors were preincubated 1 h with the indicated doses of compounds. Donor and target cells were then mixed at a 1:2 ratio in 96-well plates at a final a concentration of 1.5 × 10^6^/ml in 200 μl, in duplicates. After 48 or 72 h, cells were stained for intracellular Gag (KC57 mAb, Coulter) and analyzed by flow cytometry. When used, mCD4 or mCD4-conjugates were added 1 h before coculture. Measurement of the levels of Env epitopes at the surface of infected cells with 3BNC117 was performed by flow cytometry as previously described^62^.

## Additional Information

**How to cite this article**: Ariën, K. K. *et al*. CD4-mimetic sulfopeptide conjugates display sub-nanomolar anti-HIV-1 activity and protect macaques against a SHIV162P3 vaginal challenge. *Sci. Rep.* **6**, 34829; doi: 10.1038/srep34829 (2016).

## Supplementary Material

Supplementary Information

## Figures and Tables

**Table 1:** Antiviral activity of mCD4.1, mCD4.1-HS_12_ and mCD4.1-PS1.

Viral strains	Clade-tropism	mCD4.1 (nM)	mCD4.1-HS_12_ (nM)	mCD4.1-PS1 (nM)
VI820	A-X4R5	1686	34	25
Bal	B-R5	237	90	0.54
IIIB	B-X4	29	0.05	0.013
MN	B-X4	20	1.2	0.09
SF162	B-R5	130	10	0.11
SHIV162P3	B-R5	5372	706	36
pREJO.c/2864 cl2	T/F B-R5	108	18	0.66
pTHRO.c/2626	T/F B-R5	1181	689	91
pWITO.c/2474	T/F B-R5	1116	124	6.9
VI829	C-R5	>10000	829	16
p246F10	T/F C-R5	>10000	874	723
pZM247Fv2	T/F C-R5	>10000	>1000	3696
VI824	D-R5	3176	282	40
VI1888 (CRF01)	AE-R5	>10000	>1000	1102
Ca10-3 (CRF01)	AE-X4	96	9	0.57
MP568 (CRF02)	AG-R5	7931	879	310
CC_50_		45525	>9000	>37500

Replication competent HIV-1 viruses and SHIV162P3 were incubated with a range of concentrations of the mCD4.1 derived compounds and TZM-bl cells for 48 h. Infection was determined by luciferase activity, from which IC_50_ (expressed in nM) was calculated in GraphPad Prism 5.03 using non-linear regression. Values are means of two experiments. (T/F transmitted founder virus; X4: CXCR4 tropic; R5: CCR5 tropic, IC_50_: 50% inhibitory concentration; CC_50_: 50% cytotoxic concentration).

**Table 2:** Antiviral activity of mCD4.2, mCD4.3 and mCD4-conjugates in comparison with mAb VRC01, b12 and the NNRTI dapivirine.

Viral strains	Clade-tropism	mCD4.2 (nM)	mCD4.2 PS1(nM)	mCD4.3 (nM)	mCD4.3PS1 (nM)	mAb VRC01 (nM)	mAb b12 (nM)	TMC120 Dapivirine (nM)
VI820	A-X4R5	6.1	0.031	4.2	0.024	12	>100	2
Bal	B-R5	2	0.035	2.3	0.031	0.64	1	1.7
IIIB	B-X4	0.22	0.0073	0.16	0.0025	0.64	0.09	0.84
MN	B-X4	0.59	0.046	0.37	0.026	2	1.5	1.5
SF162	B-R5	1.3	0.01	0.66	0.0075	5.2	0.36	1.3
SHIV162P3	B-R5	69	2.2	29	1.4	8.2	6	>1000
pREJO.c/2864 cl2	T/F B-R5	76	0.28	46	0.28	0.77	>100	1.6
pTHRO.c/2626	T/F B-R5	19	8.9	15	6.4	>100	35	1.9
pWITO.c/2474	T/F B-R5	184	1.3	178	1.5	2	>100	1.1
VI829	C-R5	512	4	221	3.6	9.3	>100	1.3
P246F10	T/F C-R5	718	58	510	33	85	>100	1.4
pZM247Fv2	T/F C-R5	>1000	206	>1000	192	5.7	>100	2.3
VI824	D-R5	56	0.68	39	0.7	>100	>100	1.2
VI1888 (CRF01)	AE-R5	5382	1064	4509	1212	16	80	2.1
Ca10-3 (CRF01)	AE-X4	4.7	0.12	3.1	0.076	13	>100	1.2
MP568 (CRF02)	AG-R5	155	4.9	204	4.2	54	>100	1.1
CC50		24036	>50000	15540	>50000	>100	>300	2524

Various replication competent HIV-1 or SHIV162P3 viruses at 10^−3^ MOI were incubated with a range of concentrations of the indicated compounds and TZM-bl cells for 48 h. Infection was determined as explained in the methods section. IC_50_ values (expressed in nM) represent the mean of at least 2 independent experiments, each with triplicate measurements and were calculated in GraphPad Prism 5.03 using non-linear regression. (T/F transmitted founder virus; X4: CXCR4 tropic; R5: CCR5 tropic, IC_50_: 50% inhibitory concentration; CC_50_: 50% cytotoxic concentration).

## References

[b1] Barre-Sinoussi F. . Isolation of a T-lymphotropic retrovirus from a patient at risk for acquired immune deficiency syndrome (AIDS). Science (New York, NY) 220, 868–871 (1983). 10.1126/science.6189183 6189183

[b2] Shattock R. J. & Moore J. P. Inhibiting sexual transmission of HIV-1 infection. Nature reviews. Microbiology 1, 25–34, 10.1038/nrmicro729 (2003). 15040177

[b3] Haase A. T. Early events in sexual transmission of HIV and SIV and opportunities for interventions. Annual review of medicine 62, 127–139, 10.1146/annurev-med-080709-124959 (2011). 21054171

[b4] Bomsel M. & Alfsen A. Entry of viruses through the epithelial barrier: pathogenic trickery. Nature reviews. Molecular cell biology 4, 57–68, 10.1038/nrm1005 (2003). 12511869 PMC7097689

[b5] Burgener A., McGowan I. & Klatt N. R. HIV and mucosal barrier interactions: consequences for transmission and pathogenesis. Current opinion in immunology 36, 22–30, 10.1016/j.coi.2015.06.004 (2015). 26151777

[b6] Cicala C., Arthos J. & Fauci A. S. HIV-1 envelope, integrins and co-receptor use in mucosal transmission of HIV. Journal of translational medicine 9 Suppl 1, S2, 10.1186/1479-5876-9-s1-s2 (2011). 21284901 PMC3105502

[b7] Connell B. J. & Lortat-Jacob H. Human immunodeficiency virus and heparan sulfate: from attachment to entry inhibition. Frontiers in immunology 4, 385, 10.3389/fimmu.2013.00385 (2013). 24312095 PMC3834540

[b8] Arien K. K., Jespers V. & Vanham G. HIV sexual transmission and microbicides. Reviews in medical virology 21, 110–133, 10.1002/rmv.684 (2011). 21412935

[b9] Hladik F. & Doncel G. F. Preventing mucosal HIV transmission with topical microbicides: challenges and opportunities. Antiviral research 88 Suppl 1, S3–9, 10.1016/j.antiviral.2010.09.011 (2010). 21109065 PMC3032991

[b10] Karim Q. A. & Baxter C. Microbicides for the prevention of sexually transmitted HIV infection. Expert review of anti-infective therapy 11, 13–23, 10.1586/eri.12.153 (2013). 23428099

[b11] Klasse P. J., Shattock R. & Moore J. P. Antiretroviral drug-based microbicides to prevent HIV-1 sexual transmission. Annual review of medicine 59, 455–471, 10.1146/annurev.med.59.061206.112737 (2008). 17892435

[b12] Pirrone V., Wigdahl B. & Krebs F. C. The rise and fall of polyanionic inhibitors of the human immunodeficiency virus type 1. Antiviral research 90, 168–182, 10.1016/j.antiviral.2011.03.176 (2011). 21439325

[b13] Romano J. W., Robbiani M., Doncel G. F. & Moench T. Non-specific microbicide product development: then and now. Current HIV research 10, 9–18 (2012). 22264041 10.2174/157016212799304625 PMC4391741

[b14] Tan S. . Polyanionic candidate microbicides accelerate the formation of semen-derived amyloid fibrils to enhance HIV-1 infection. PloS one 8, e59777, 10.1371/journal.pone.0059777 (2013). 23544097 PMC3609764

[b15] Van Damme L. . Lack of effectiveness of cellulose sulfate gel for the prevention of vaginal HIV transmission. The New England journal of medicine 359, 463–472, 10.1056/NEJMoa0707957 (2008). 18669425

[b16] Moog C. . Protective effect of vaginal application of neutralizing and nonneutralizing inhibitory antibodies against vaginal SHIV challenge in macaques. Mucosal immunology 7, 46–56, 10.1038/mi.2013.23 (2014). 23591718

[b17] Pegu A. . Neutralizing antibodies to HIV-1 envelope protect more effectively *in vivo* than those to the CD4 receptor. Science translational medicine 6, 243ra288, 10.1126/scitranslmed.3008992 (2014). PMC4562469 24990883

[b18] Baeten J. M. . Use of a Vaginal Ring Containing Dapivirine for HIV-1 Prevention in Women. The New England journal of medicine, 10.1056/NEJMoa1506110 (2016). PMC4993693 26900902

[b19] Sokal D. C. . Safety of tenofovir gel, a vaginal microbicide, in South African women: results of the CAPRISA 004 Trial. Antiviral therapy 18, 301–310, 10.3851/imp2311 (2013). 22914267

[b20] Malcolm R. K. . Pharmacokinetics and efficacy of a vaginally administered maraviroc gel in rhesus macaques. The Journal of antimicrobial chemotherapy 68, 678–683, 10.1093/jac/dks422 (2013). 23111849 PMC3566668

[b21] Chen B. A. . Phase 1 Safety, Pharmacokinetics, and Pharmacodynamics of Dapivirine and Maraviroc Vaginal Rings: A Double-Blind Randomized Trial. Journal of acquired immune deficiency syndromes (1999) 70, 242–249, 10.1097/qai.0000000000000702 (2015). 26034880 PMC4607587

[b22] Baleux F. . A synthetic CD4-heparan sulfate glycoconjugate inhibits CCR5 and CXCR4 HIV-1 attachment and entry. Nat Chem Biol 5, 743–748 (2009). 19734912 10.1038/nchembio.207

[b23] Connell B. J. . A synthetic heparan sulfate-mimetic peptide conjugated to a mini CD4 displays very high anti- HIV-1 activity independently of coreceptor usage. Chemistry & biology 19, 131–139, 10.1016/j.chembiol.2011.12.009 (2012). 22284360

[b24] Crublet E., Andrieu J. P., Vives R. R. & Lortat-Jacob H. The HIV-1 envelope glycoprotein gp120 features four heparan sulfate binding domains, including the co-receptor binding site. J Biol Chem 283, 15193–15200 (2008). 18378683 10.1074/jbc.M800066200 PMC3258890

[b25] Vives R. R., Imberty A., Sattentau Q. J. & Lortat-Jacob H. Heparan sulfate targets the HIV-1 envelope glycoprotein gp120 coreceptor binding site. J Biol Chem 280, 21353–21357 (2005). 15797855 10.1074/jbc.M500911200

[b26] Farzan M. . Tyrosine sulfation of the amino terminus of CCR5 facilitates HIV-1 entry. Cell 96, 667–676 (1999). 10089882 10.1016/s0092-8674(00)80577-2

[b27] Keele B. F. . Identification and characterization of transmitted and early founder virus envelopes in primary HIV-1 infection. Proceedings of the National Academy of Sciences of the United States of America 105, 7552–7557, 10.1073/pnas.0802203105 (2008). 18490657 PMC2387184

[b28] Marx P. A. . Progesterone implants enhance SIV vaginal transmission and early virus load. Nature medicine 2, 1084–1089 (1996). 10.1038/nm1096-1084 8837605

[b29] Aarnink A. . Influence of the MHC genotype on the progression of experimental SIV infection in the Mauritian cynomolgus macaque. Immunogenetics 63, 267–274, 10.1007/s00251-010-0504-6 (2011). 21234560

[b30] Dereuddre-Bosquet N. . MiniCD4 microbicide prevents HIV infection of human mucosal explants and vaginal transmission of SHIV(162P3) in cynomolgus macaques. PLoS pathogens 8, e1003071, 10.1371/journal.ppat.1003071 (2012). 23236282 PMC3516572

[b31] Lederman M. M. . Prevention of vaginal SHIV transmission in rhesus macaques through inhibition of CCR5. Science (New York, NY) 306, 485–487, 10.1126/science.1099288 (2004). 15486300

[b32] Tsai C. C. . Cyanovirin-N inhibits AIDS virus infections in vaginal transmission models. AIDS research and human retroviruses 20, 11–18, 10.1089/088922204322749459 (2004). 15000694

[b33] Veazey R. S. . Protection of rhesus macaques from vaginal infection by vaginally delivered maraviroc, an inhibitor of HIV-1 entry via the CCR5 co-receptor. The Journal of infectious diseases 202, 739–744, 10.1086/655661 (2010). 20629537 PMC2916941

[b34] Veazey R. S. . Protection of macaques from vaginal SHIV challenge by vaginally delivered inhibitors of virus-cell fusion. Nature 438, 99–102, 10.1038/nature04055 (2005). 16258536

[b35] Grupping K. . MiniCD4 protein resistance mutations affect binding to the HIV-1 gp120 CD4 binding site and decrease entry efficiency. Retrovirology 9, 36, 10.1186/1742-4690-9-36 (2012). 22551420 PMC3408336

[b36] Morellato-Castillo L. . Interfacial cavity filling to optimize CD4-mimetic miniprotein interactions with HIV-1 surface glycoprotein. Journal of medicinal chemistry 56, 5033–5047, 10.1021/jm4002988 (2013). 23710622 PMC3812931

[b37] Caskey M. . Viraemia suppressed in HIV-1-infected humans by broadly neutralizing antibody 3BNC117. Nature 522, 487–491, 10.1038/nature14411 (2015). 25855300 PMC4890714

[b38] Arien K. K. . Diaryltriazine non-nucleoside reverse transcriptase inhibitors are potent candidates for pre-exposure prophylaxis in the prevention of sexual HIV transmission. The Journal of antimicrobial chemotherapy 68, 2038–2047, 10.1093/jac/dkt166 (2013). 23645585

[b39] Selhorst P. . Human immunodeficiency virus type 1 resistance or cross-resistance to nonnucleoside reverse transcriptase inhibitors currently under development as microbicides. Antimicrobial agents and chemotherapy 55, 1403–1413, 10.1128/aac.01426-10 (2011). 21282453 PMC3067143

[b40] Schader S. M. . HIV gp120 H375 is unique to HIV-1 subtype CRF01_AE and confers strong resistance to the entry inhibitor BMS-599793, a candidate microbicide drug. Antimicrobial agents and chemotherapy 56, 4257–4267, 10.1128/aac.00639-12 (2012). 22615295 PMC3421599

[b41] Murooka T. T. . HIV-infected T cells are migratory vehicles for viral dissemination. Nature 490, 283–287, 10.1038/nature11398 (2012). 22854780 PMC3470742

[b42] Sattentau Q. J. The direct passage of animal viruses between cells. Current opinion in virology 1, 396–402, 10.1016/j.coviro.2011.09.004 (2011). 22440841

[b43] Sourisseau M., Sol-Foulon N., Porrot F., Blanchet F. & Schwartz O. Inefficient human immunodeficiency virus replication in mobile lymphocytes. Journal of virology 81, 1000–1012, 10.1128/jvi.01629-06 (2007). 17079292 PMC1797449

[b44] Malbec M. . Broadly neutralizing antibodies that inhibit HIV-1 cell to cell transmission. The Journal of experimental medicine 210, 2813–2821, 10.1084/jem.20131244 (2013). 24277152 PMC3865481

[b45] Huang C. C. . Structural basis of tyrosine sulfation and VH-gene usage in antibodies that recognize the HIV type 1 coreceptor-binding site on gp120. Proceedings of the National Academy of Sciences of the United States of America 101, 2706–2711 (2004). 14981267 10.1073/pnas.0308527100 PMC365685

[b46] Andrews G. P. . Characterization of the rheological, mucoadhesive, and drug release properties of highly structured gel platforms for intravaginal drug delivery. Biomacromolecules 10, 2427–2435, 10.1021/bm9003332 (2009). 19642670 PMC2745825

[b47] Veazey R. S. . Topically applied recombinant chemokine analogues fully protect macaques from vaginal simian-human immunodeficiency virus challenge. The Journal of infectious diseases 199, 1525–1527, 10.1086/598685 (2009). 19331577 PMC3626081

[b48] Ramirez Valdez K. P. . Complementary and synergistic activities of anti-V3, CD4bs and CD4i antibodies derived from a single individual can cover a wide range of HIV-1 strains. Virology 475, 187–203, 10.1016/j.virol.2014.11.011 (2015). 25486586

[b49] Amadi B. . Reduced production of sulfated glycosaminoglycans occurs in Zambian children with kwashiorkor but not marasmus. The American journal of clinical nutrition 89, 592–600, 10.3945/ajcn.2008.27092 (2009). 19116330

[b50] Ceballos A. . Spermatozoa capture HIV-1 through heparan sulfate and efficiently transmit the virus to dendritic cells. The Journal of experimental medicine 206, 2717–2733, 10.1084/jem.20091579 (2009). 19858326 PMC2806607

[b51] Dale B. M., Alvarez R. A. & Chen B. K. Mechanisms of enhanced HIV spread through T-cell virological synapses. Immunological reviews 251, 113–124, 10.1111/imr.12022 (2013). 23278744

[b52] Abela I. A. . Cell-cell transmission enables HIV-1 to evade inhibition by potent CD4bs directed antibodies. PLoS pathogens 8, e1002634, 10.1371/journal.ppat.1002634 (2012). 22496655 PMC3320602

[b53] Sigal A. . Cell-to-cell spread of HIV permits ongoing replication despite antiretroviral therapy. Nature 477, 95–98, 10.1038/nature10347 (2011). 21849975

[b54] Barouch D. H. . Therapeutic efficacy of potent neutralizing HIV-1-specific monoclonal antibodies in SHIV-infected rhesus monkeys. Nature 503, 224–228, 10.1038/nature12744 (2013). 24172905 PMC4017780

[b55] Shingai M. . Passive transfer of modest titers of potent and broadly neutralizing anti-HIV monoclonal antibodies block SHIV infection in macaques. The Journal of experimental medicine 211, 2061–2074, 10.1084/jem.20132494 (2014). 25155019 PMC4172223

[b56] Schwartz J. A. . An HIV gp120-CD4 Immunogen Does Not Elicit Autoimmune Antibody Responses in Cynomolgus Macaques. Clinical and vaccine immunology : CVI 23, 618–627, 10.1128/cvi.00115-16 (2016). 27193040 PMC4933776

[b57] Harouse J. M. . Mucosal transmission and induction of simian AIDS by CCR5-specific simian/human immunodeficiency virus SHIV(SF162P3). Journal of virology 75, 1990–1995, 10.1128/jvi.75.4.1990-1995.2001 (2001). 11160699 PMC115146

[b58] Harouse J. M., Gettie A., Tan R. C., Blanchard J. & Cheng-Mayer C. Distinct pathogenic sequela in rhesus macaques infected with CCR5 or CXCR4 utilizing SHIVs. Science (New York, NY) 284, 816–819 (1999). 10.1126/science.284.5415.816 10221916

[b59] Bourry O. . Prevention of vaginal simian immunodeficiency virus transmission in macaques by postexposure prophylaxis with zidovudine, lamivudine and indinavir. AIDS (London, England) 23, 447–454, 10.1097/QAD.0b013e328321302d (2009). 19240457

[b60] Mannioui A. . Dynamics of viral replication in blood and lymphoid tissues during SIVmac251 infection of macaques. Retrovirology 6, 106, 10.1186/1742-4690-6-106 (2009). 19930655 PMC2789052

[b61] Lepelley A. . Innate sensing of HIV-infected cells. PLoS pathogens 7, e1001284, 10.1371/journal.ppat.1001284 (2011). 21379343 PMC3040675

[b62] Bruel T. . Elimination of HIV-1-infected cells by broadly neutralizing antibodies. Nature communications 7, 10844, 10.1038/ncomms10844 (2016). PMC4782064 26936020

