# Supplementary material for: CD4-mimetic sulfopeptide conjugates display sub-nanomolar anti-HIV-1 activity and protect macaques against a SHIV162P3 vaginal challenge
Source: Sci Rep. 2016 Oct 10;6:34829. doi: 10.1038/srep34829 (PMC5056392; doi:10.1038/srep34829)

CD4-mimetic sulfopeptide conjugates display sub nanomolar anti-HIV-1 activity and protect macaques against a SHIV162P3 vaginal challenge

Kevin K. Ariën[1*], Françoise Baleux[2*], Delphine Desjardins[3,4,5], Françoise Porrot[6], Yves-Marie Coic[2], Jo Michiels[1], Kawthar Bouchemal[7], David Bonnaffé[8], Olivier Schwartz[6], Roger Le Grand[3,4,5], Guido Vanham[1,9], Nathalie Dereuddre-Bosquet[3,4,5], Hugues Lortat-Jacob[10]

Supplementary information

Figure S1 : mCD4.n-PS1 structure (n = 1, 2 or 3)

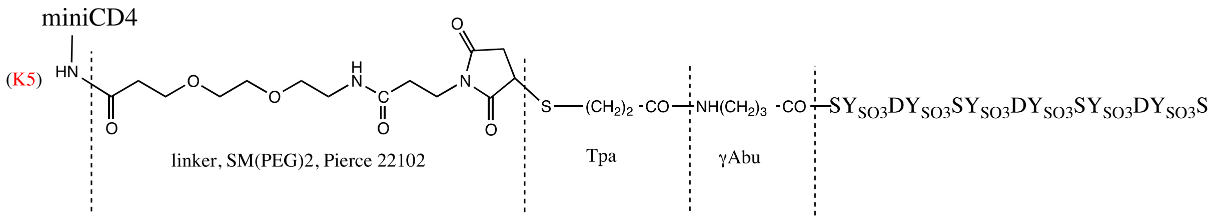

Tpa: Thiopropionic acid
γAbu: γ aminobutyric acid

Peptide	Sequence
M48U1	Tpa-NLHFCQLRC**K_{11}**SLGLLGRCApTU$_1$CACV-amide
mCD4.1	Tpa-NLH**K_5**CQLRCSSLGLLGRCAGS-Bip-CACV-amide
mCD4.2	Tpa-NLH**K_5**CQLRC**S_{11}**SLGLLGRCApTU$_1$CACV-amide
mCD4.3	Tpa-NLH**K_5**CQLRC**R_{11}**SLGLLGRCApTU$_1$CACV-amide
PS1	NH_2-$(CH_2)_3$-CO-NH-$SY_{SO3}DY_{SO3}SY_{SO3}DY_{SO3}SY_{SO3}DY_{SO3}S$
γAbu	NH_2-$(CH_2)_3$-CO

A single Lysine residue was introduced at position 5 of the sequences to be synthetized in order to correctly orient PS1 toward the CD4i epitopes {Baleux, 2009 #53}. M48U1 Lys11 was replaced by a Ser residue to obtain a miniCD4 peptide bearing a single amino group for PS1 derivatization (mCD4.2). As removing a positive charge in the sequence could potentially affects gp120 miniCD4 affinity and antiviral activity, an Arg residue was introduced at position 11 leading to mCD4.3 peptide. To confirm the superiority of K5 PS1 coupling versus K11, M48U1-PS1 was also synthesized and the anti-viral activity of the two compounds were compared (Supplementary Table I).

Supplementary Table SI

Viral strain	Clade-tropism	M48U1-PS1 (K11 coupling)	mCD4.2-PS1 (K5 coupling)	activity fold increase
**Bal**	**B-R5**	0.13	0.035	3.8
**IIIB**	**B-X4**	0.02	0.007	2.8

Antiviral activity of M48U1-PS1 and mCD4.2-PS1: Replication competent HIV-1 (Bal or IIIB) was incubated with a range of concentrations of the compounds and TZM-bl cells for 48h. Infection was determined and the corresponding IC_{50} (expressed in nM) was determined.

Synthesis scheme for the preparation of mCD4 and PS1 peptides

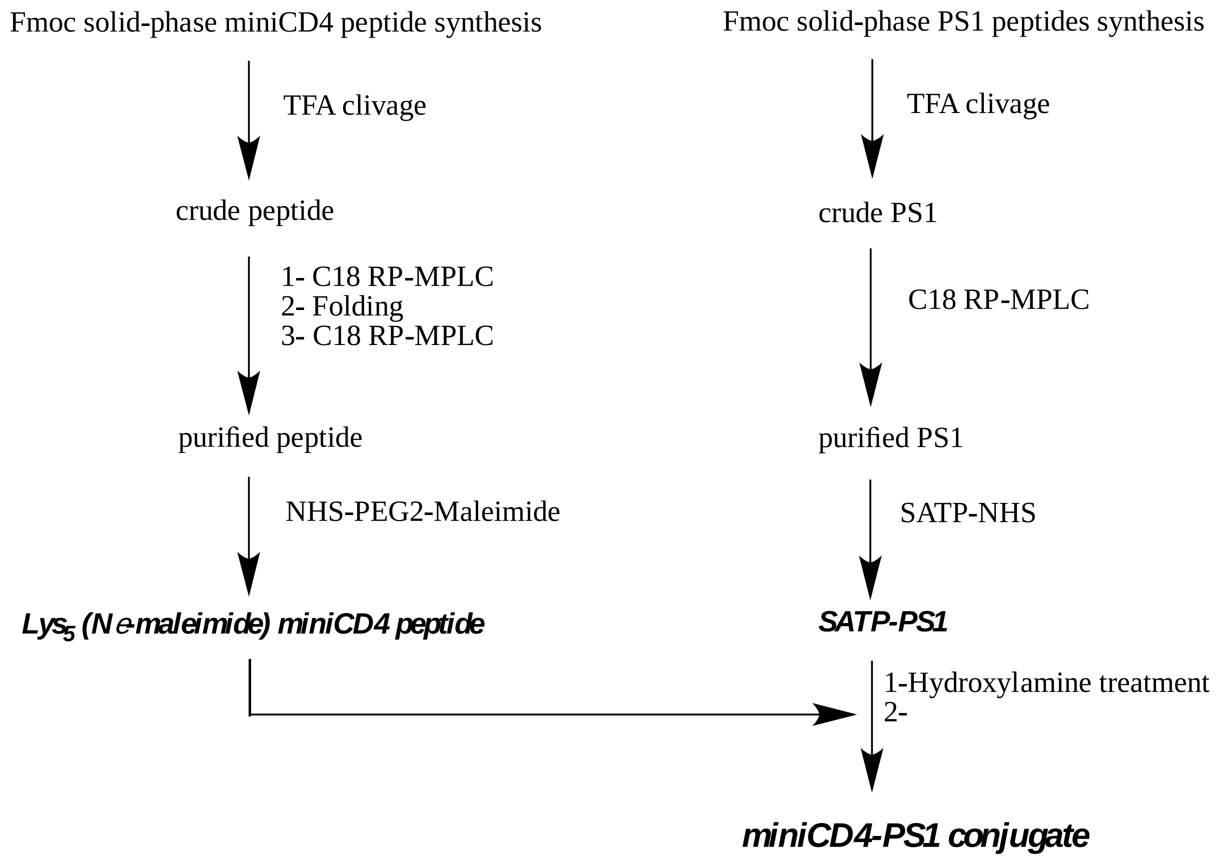

Supplementary Material and Methods

Reagents

All reagents for peptides synthesis were from Applied Biosystems/ Life Technolgies, except Fmoc-4,4'- Biphenylalanine (Bip) from Interchim, S-trityl Thiopropionic acid (Tpa) and Fmoc-4-cyclohexylmethoxy-L-phenylalanine (U1) from Iris Biotech GmbH, Fmoc-γAminobutyric acid (γAbu), Fmoc-D-Proline and Fmoc-L-O-sulfo-Tyrosine tetrabutylammonium salt, pseudoproline dipeptides Fmoc-Ser(tBu)-Ser($\psi^{Me,Me}$Pro) and FmocGly- Ser($\psi^{Me,Me}$Pro) from Novabiochem. Succinimidyl-[(*N*-maleimidopropionamido)-diethyleneglycol] ester (NHS-PEG_2-Maleimide) and SATP-NHS (*N*-Succinimidyl S-Acetylthiopropionate) were from Pierce/Thermo Scientific. PS 2-CT-Ser (tBu) was from Rapp polymere GmbH.

All peptides and conjugates were purified and controlled by HPLC, mass spectrometry (ESI-MS on Q-Tof micro Waters). Synthesis yields were calculated from final isolated peptides after quantification by amino acids analysis on a Hitachi L8800 apparatus.

General protocol for mCD4 peptides synthesis

mCD4 peptides were synthesized on a Rink Amide resin (100 µmoles) using Fmoc chemistry and HATU/DIEA activation for Fmoc amino acids coupling (1 mmole, 10 equiv.) on an ABI433 apparatus. For mCD4.1, Ser11Ser12 and GLy21Ser22 were introduced as pseudoproline dipeptides. After chain elongation, peptides were released from the resin by TFA/H2O/Thioanisole/Phenol/Ethanedithiol/Triisopropyl silane (82/5/5/5/2/1), 20 ml, 2:30 h treatment. Precipitation in cold diethylether afforded the crude peptides that were collected by centrifugation and dried under vacuum. The precipitated peptides were solubilized by addition of 10 ml pure AcOH, followed by 40 ml of water (final concentration: 6 mg/ml) and placed for 45 min in Ultrasonic bath then freeze-dried. Crude peptides (12 mg/ml in AcOH/CH_3CN/H2O, 12/7/71) were purified by C18 Reverse-Phase Medium Pressure Chromatography (C18 RP-MPLC, 30 mm X 340 mm column) using a 20-80 linear gradient of acetonitrile in 0.08% aqueous TFA over 60 minutes at 25 ml flow rate. Purified SH peptides were dissolved in 65 ml of water (2 mg/ml) then dilute with 65 ml of 0.1 M pH 8.5 TRIS buffer (final concentration: 1 mg/ml). Folding was performed by sequential addition of GSH and GSSG (20 mM/2 mM final concentration) and was followed by analytical RP-HPLC (AERIS peptide XB-C18, Phenomenex, 3.6 µm, 100 x 2.1 mm, TFA 0.08%/CH3CN gradient). Folding was complete after 45 minutes. After acidification of the reaction mixture by 1 ml of pure

AcOH, folded mCD4 peptides were isolated by RP-MPLC using a 0-60 linear gradient with the same eluents as above.

	**Isolated yield (%)**	**Expected [M+H]$^+$ (monoisotopic)**	**Found [M+H]$^+$**
**mCD4.1**	21	$C_{122}H_{194}N_{38}O_{32}S_6$: 2896.3124	2896.4170
**M48U1**	6	$C_{133}H_{212}N_{38}O_{32}S_6$: 2989.4169	2986.4021
**mCD4.2**	15	$C_{127}H_{208}N_{38}O_{33}S_6$: 2986.4169	2986.4021
**mCD4.3**	21	$C_{130}H_{215}N_{41}O_{32}S_6$: 3055.4259	3055.4546

Lys5 (Nε-maleimide) mCD4 peptides

To mCD4 peptide water solution (2 mg/ml) was slowly added under mild agitation to avoid foaming 100 mM sodium phosphate buffer pH 7.2 to reach a 10 mM final buffer concentration. NHS-PEG2-Maleimide (10 molar equivalents) in DMSO was drop-wise added to the peptide solution. After 10 minutes, the reaction mixture was acidified by pure AcOH (4% final concentration) and purified by C18 RP-MPLC using the same conditions as for mCD4 peptide.

	**Isolated yield (%)**	**Expected [M+H]$^+$ (monoisotopic)**	**Found [M+H]$^+$**
**mCD4.1Mal**	77	$C_{136}H_{212}N_{40}O_{38}S_6$: 3206.4289	3206.6572
**M48U1Mal**	80	$C_{147}H_{230}N_{40}O_{38}S_6$: 3356.5697	3356.4504
**mCD4.2Mal**	60	$C_{141}H_{226}N_{40}O_{39}S_6$: 3296.5333	3296.4609
**mCD4.3Mal**	66	$C_{144}H_{233}N_{43}O_{38}S_6$: 3365.6024	3365.7483

Polyanionic peptide PS1

PS1 peptide was synthesized on a Serine preloaded Chlorotrityl resin, PS 2-CT-Ser (tBu), (100 µmoles) using Fmoc chemistry and HATU/DIEA activation for Fmoc amino acids coupling (1 mmole, 10 equiv.). Tyrosine sulfate was incorporated using Fmoc-L-O-sulfo-Tyrosine tetrabutylammonium salt. γAminobutyric acid was introduced at the N-terminus of the peptide chain using standard amino acid coupling protocol. In order to maintain the sulfate group on the tyrosine residues, all the cleavage protocol was performed at 4°C (ice bath). After 1h30 in TFA/TIS/H2O 95/2.5/2.5 (9 ml), precipitation in cold diethylether afforded the crude peptide that was collected by centrifugation, dried under vacuum, solubilized in 30 ml of 100 mM ammonium hydrogenocarbonate and freeze-dried. Crude peptide (237 mg in 15 ml of 100 mM triethylamine acetate buffer) was purified by C18 RP-MPLC using a 0-60 linear gradient of acetonitrile in 100 mM triethylacetate buffer over 60 minutes. After

freeze-drying, the purified peptide was solubilized in 100 ml H2O and freeze dried (3 times to remove excess of triethylamine acetate). Yield: 83 mg. PS1 was controlled by MS using negative mode.

	**Isolated yield (%)**	**Expected [M-H]$^-$ (monoisotopic)**	**Found [M-H]$^-$**
**PS1**	37	$C_{82}H_{98}N_{14}O_{49}S_6$: 2253.3811	2253.3853

SATP-PS1 peptide

To 100 mg of PS1 peptide in 12 ml of 0.1 M sodium phosphate buffer pH 7.2 was drop wise added 40 mg of SATP-NHS (6 molar equivalents) in 300 µl of DMSO. After 40 minutes, the reaction medium was injected onto C18 RP-MPLC using the same conditions as for PS1.

SATP-PS1 was controlled by MS using negative mode. Yield: 80 mg

	**Isolated yield (%)**	**Expected [M-H]$^-$ (monoisotopic)**	**Found [M-H]$^-$**
**SATP-PS1**	76	$C_{87}H_{104}N_{14}O_{51}S_7$: 2383.3942	2383.3174

General protocol for miniCD4-PS1 peptides synthesis

SATP-PS1 peptide (109 mg) was dissolved in 20 ml of 100 mM sodium phosphate buffer pH 7.2. Two ml of a solution containing 0.5 M Hydroxylamine chlorhydrate in 0.1 M sodium phosphate (pH adjusted to 7.2 by 4N NaOH) were added. After 45 minutes, 52 mg of Lys5 (Nε-maleimide) mCD4.1 peptide in 22 ml of water were added. After 35 minutes, mCD4.1-PS1 conjugate was purified by C18 RP-MPLC using the same conditions as for PS1.

Final purity was controlled by analytical C18 RP-HPLC (AERIS peptide XB-C18, Phenomenex, 3.6 µm, 100 x 2.1 mm) using a 20-40 linear gradient of acetonitrile in 100 mM aqueous triethylamine acetate buffer over 20 min at 0.35 ml/min flow rate. Yield: 48 mg (data for mCD4.1-PS1). mCD4.2-PS1 and mCD4.3-PS1 were prepared using the same protocol. All conjugates were controlled by ESI-MS using negative mode.

	**Isolated yield (%)**	**Expected M (average)**	**Found M**
**mCD4.1-PS1**	64	$C_{221}H_{314}N_{54}O_{88}S_{13}$: 5552.0933	5552.1108
**M48U1-PS1**	92	$C_{232}H_{332}N_{54}O_{88}S_{13}$: 5702.3572	5702.9180
**mCD4.2-PS1**	66	$C_{226}H_{328}N_{54}O_{89}S_{13}$: 5642.2589	5642.6030
**mCD4.3-PS1**	66	$C_{229}H_{335}N_{57}O_{88}S_{13}$: 5705.9782	5705.9243

ESI-MS spectra of mCD4.1-PS1

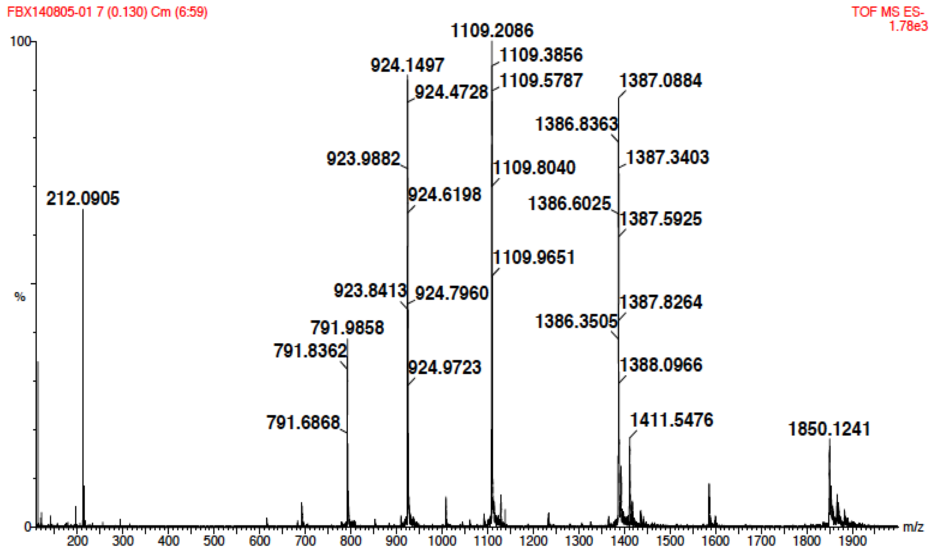

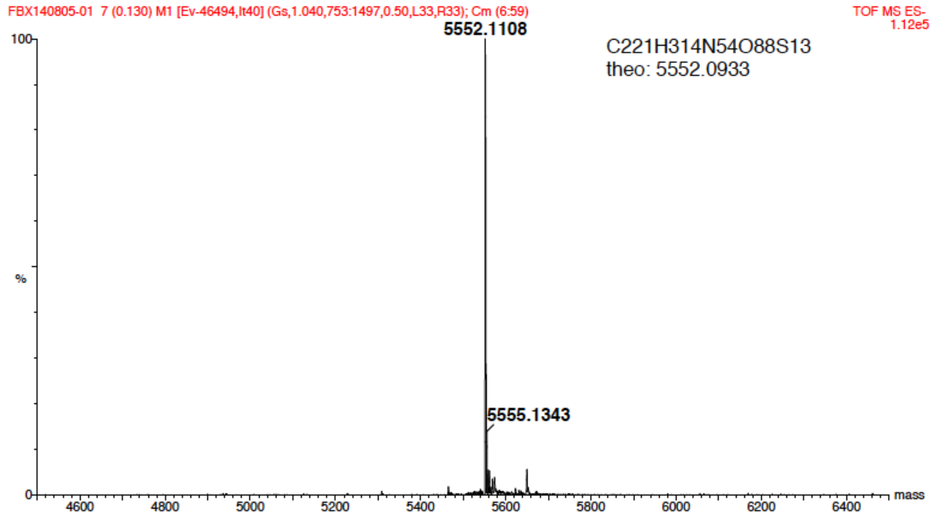

Supplement: Supplementary Information [file srep34829-s1.pdf]
